# Differential Effects of Adding Graphene Nanoplatelets on the Mechanical Properties and Crystalline Behavior of Polypropylene Composites Reinforced with Carbon Fiber or Glass Fiber

**DOI:** 10.3390/ma18050926

**Published:** 2025-02-20

**Authors:** Hiroki Satoh, Ayumu Morita, Yoshihiko Arao

**Affiliations:** 1 Department of Applied Mechanics and Aerospace Engineering, School of Fundamental Science and Engineering, Waseda University, 3-4-1, Okubo, Shinjuku-ku, Tokyo 169-8555, Japan; hiroki1219@toki.waseda.jp (H.S.); ayumu0201@toki.waseda.jp (A.M.); 2 Kagami Memorial Research Institute for Materials Science and Technology, Waseda University, 2-8-26, Nishiwaseda, Shinjuku-ku, Tokyo 169-0051, Japan

**Keywords:** carbon fiber, glass fiber, graphene nanoplatelets, polypropylene, hybrid composites, injection molding, mechanical properties, crystallization

## Abstract

Short fiber-reinforced thermoplastic composites (SFRTPs) have excellent recyclability and processability, but their mechanical properties are weak compared to continuous fiber products. Various studies have reported that the addition of GNPs improves the mechanical properties of SFRTPs, but it is unclear what effect different types of reinforcing fibers have on a hybrid composite system. In this study, the effect of adding a small amount (1 wt%) of graphene nanoplatelets (GNPs) to fiber-reinforced polypropylene composites on their mechanical properties was investigated from a crystallinity perspective. GNPs were mixed with polypropylene (PP)/carbon fiber (CF) or PP/glass fiber (GF) using a melt blending process, and composites were molded by injection molding. The results of mechanical property characterization showed no significant effect when GNPs were added to PP/CF, but when GNPs were added to PP/GF, this increased the composite’s tensile strength and Young’s modulus by approximately 20% and 10%, respectively. The interfacial shear strength (IFSS) predicted using the modified Kelly–Tyson equation did not change much before and after the addition of GNPs to PP/CF. On the other hand, the IFSS increased from 10.8 MPa to 19.2 MPa with the addition of GNPs to PP/GF. The increase in IFSS led to an increase in the tensile strength of PP/GF with the incorporation of GNPs. Differential scanning calorimetry (DSC) indicated that GNPs accelerated the crystallization rate, and the X-ray diffraction (XRD) results confirmed that GNPs acted as a crystal nucleating agent. However, CF was also shown to be a nucleating agent, limiting the effect of GNP addition. In other words, it can be said that the addition of GNPs to PP/GF is more effective than their addition to PP/CF due to the differential crystallization effects of each fiber.

## 1. Introduction

Polypropylene (PP) is a kind of commodity polymer and is used in a wide range of fields, including daily necessities, home appliances, and automotive parts, because of its low cost, low density, and easy processability [1,2,3]. PP lacks strength and stiffness, limiting its applicability. Therefore, many studies have incorporated reinforcing fillers such as graphene [4,5,6], carbon nanotubes (CNTs) [2,7], clay [8,9,10,11], silica [12], and cellulose [13] into the PP matrix to improve its mechanical properties. The incorporation of fibers such as carbon fibers (CFs) [14,15,16], glass fibers (GFs) [16,17], and aramid fibers (AFs) [18] also greatly aids in improving the mechanical properties of composites. Fiber-reinforced plastics (FRPs) using PP as the base material have superior recyclability, corrosion resistance, and durability compared to conventional composite materials using thermosetting plastics [19,20,21,22,23]. Xian et al. [23] performed durability tests on glass fiber-reinforced PP (GFRPP) plates using a combination of water immersion and bending loading. Under certain conditions, GFRPP was shown to have a close or higher tensile strength retention rate and service life compared to thermosetting plastics. Achukwu et al. [24] investigated the effects of the extrusion and compression molding process, conducted 10 times, on the mechanical properties of GF/PP. The mechanical properties decreased with each process cycle, but the chemical bonding and elemental composition were not destroyed. This can be achieved by adding virgin PP, which reduces costs and preserves the environment. Typically, thermoplastics such as PP and polyamide (PA) are blended with short fibers. The advantage of short fiber-reinforced composites over composites reinforced with continuous fibers or long fibers is superior processability, but they have weak mechanical properties.

The strength of short fiber-reinforced plastics (SFRPs) is determined by the fiber length, fiber content, and the interfacial shear strength (IFSS) between the fiber and the matrix [25]. However, it is not easy to adjust the fiber length as it depends on the process during blending and molding. Therefore, increasing the IFSS is an effective way to increase the strength of SFRPs.

Usually, maleic anhydride-grafted polypropylene (MAPP) is added to composites to increase the IFSS between the fiber and the matrix [15]. Maleic anhydride groups grafted onto PP react with polar groups on the fiber surface to form chemical bonds and improve interfacial bonding. Yamaguchi et al. [26] investigated the effects on IFSS between CF and a PP matrix using MAPP with different amounts of MA. Although the IFSS increase rate was not proportional to the amount of MA, the IFSS increased by more than 90% no matter which MAPP was added to the PP matrix at 10 wt%. Lee et al. [27] used plasma treatment to improve the adhesion property between recycled CF and polypropylene with MAPP. They found that the IFSS increased under 0.5 s of plasma treatment. Beyond 0.5 s, the IFSS slightly decreased. However, the surface treatment or modification of fibers involves a complex process and usually causes some damage to the fibers.

It has been reported that adding small amounts of nanofiller to the matrix increases the strength of SFRPs [28]. Arao et al. [28] blended MAPP with CF/PP composites to increase their IFSS and then added small amounts (<5 wt%) of various types of nanofillers, including alumina, silica, CNTs, and clay. They concluded that the IFSS increases when nanofillers are incorporated into the matrix, with the exception of clay. CF/PP composites incorporating the nanofiller exhibited a maximum increase in tensile strength and elastic modulus of approximately 44% and 58%, respectively.

Composites reinforced with micro-scale and nano-scale fillers are called hybrid composites. Hybrid composites have better mechanical, thermal, and electrical properties than materials reinforced with fillers of either scale. Recently, graphene nanoplatelets (GNPs) have attracted attention as a nano-scale filler, and their application in composite materials is increasing due to their relatively low cost and mass production [29]. GNPs are stacked structures of graphene sheets in the form of dozens of layers produced due to van der Waals forces.

Pedrazzoli et al. [30,31,32] investigated the effect of adding GNPs (<10 wt%) in a PP matrix on GF/PP composites. Tests evaluating interfacial properties and observations of fracture surfaces indicated that the addition of GNPs improved adhesion between GF and the PP matrix. Therefore, the mechanical properties of the hybrid composite were also improved. Papageorgiou et al. [33] investigated the effect of the addition of a high GNP content (>10 wt%) on the mechanical properties and thermal conductivity of GF/PP composites and compared them to a modified rule of mixture. The flexural strength of the hybrid composite decreased, but the Young’s modulus increased. The reason for the decrease in flexural strength was agglomeration due to the high content of GNPs. The thermal conductivity of the composite was almost unaffected by the presence of GF in the PP, whereas the addition of GNPs led to an approximate fivefold increase in thermal conductivity compared to the PP matrix. Based on these results, it can be said that the amount of GNPs added must be small (<5 wt%) in order to achieve both increased stiffness and increased tensile strength in hybrid composites. Junaedi et al. [34] investigated the mechanical properties of CF/PP composites with GNPs (5 wt% or less) by tensile testing and subsequent fracture surface observations. They concluded that the incorporation of GNPs increased the interfacial adhesion and increased the Young’s modulus and tensile strength of the hybrid composite by up to approximately 27% and 20%, respectively.

It is known that the properties of crystalline polymers such as PP depend on the crystal structure, and it has been reported that crystal morphology affects IFSS and stiffness improvement [35,36,37]. Sansone et al. [37] found that the growth of PP crystals from GNPs formed a stiffness gradient from high-stiffness GF to low-stiffness PP by experimenting with the incorporation of GNPs into GF/PP composites. The interphase of the hybrid composite was twice as wide as that of PP with embedded sized GF. This interphase improves stress transfer. This effect is expected to vary depending on the type of reinforcing fiber. GF has little nucleation ability, while CF and AF can serve as nucleating agents in a PP matrix [38,39,40]. It would be very interesting to study the effects of hybrid composites incorporating GNPs as nucleating agents in SFRPs with different crystal morphologies.

In this study, we investigated the effect of adding a small amount of GNPs (1 wt%) to PP composites reinforced with different fibers on their mechanical properties and crystalline behavior. The GNP content was determined to improve both the stiffness and tensile strength of the hybrid composites. If the amount of GNPs is large, the stiffness of nanocomposites increases, but if it exceeds 1 wt%, agglomeration of GNPs occurs and the tensile strength decreases [37]. PP composites were reinforced with either GF or CF. Both fibers’ content was fixed 20 wt%. Hybrid composites were prepared by melt blending, followed by injection molding. Mechanical properties were characterized using tensile testing, crystallinity was evaluated using X-ray diffraction (XRD), and crystallization kinetics under isothermal conditions were analyzed using a differential scanning calorimeter (DSC). The crystalline morphology around fibers in PP matrix was observed using a polarizing optical microscope (POM), and the effects of different fiber types on the crystalline behavior were evaluated.

## 2. Materials and Methods

### 2.1. Materials

Injection molding-grade polypropylene (NOVATEC^TM^ PP, MA3) was purchased from Japan Polypropylene Corporation (Tokyo, Japan). Carbon fiber (T700) was supplied by Toray industries, Inc. (Tokyo, Japan). Glass fiber was offered by Nitto Boseki Co., Ltd. (Tokyo, Japan). Carbon fiber and glass fiber were used as reinforcement materials. The initial fiber length for both fibers was 3 mm. Graphene nanoplatelet (GnP M5, average particle size = 5 μm) powder was purchased from XG Science Co., Ltd. (Lansing, MI, USA). M5 was selected because it has been reported that a smaller particle size and thinner GNP thickness improve the mechanical properties of PP composites when prepared by melt blending [41,42].

### 2.2. Preparation of Composites

All samples were prepared by melt blending, followed by injection molding. The weight of each material in the composition is presented in Table 1. All blends were processed in a micro-compounder (Xplore, Sittard, The Netherlands MC 15HT) at 240 °C and 100 rpm for 5 min. After melt blending, dumbbell-shaped specimens (JIS K7161-2/1BA) were immediately obtained using an injection molding machine (Xplore, IM 12). The thickness and width of the specimens were 2 and 5 mm, respectively.

### 2.3. Sample Characterization

The tensile mechanical properties of the specimens were measured using a tensile testing machine (AG-25TB, SHIMAZDU, Kyoto, Japan) at a crosshead speed of 1 mm/min. Each specimen was measured using 5 samples. An extensometer device (SG-10-50, SHIMADZU) with a gauge length of 10 mm was used to measure strain during the tensile test.

Isothermal characterization of the composites was carried out using differential scanning calorimetry (DSC, DSC8500, Perkin Elmer, Waltham, MA, USA). Samples were heated from room temperature to 240 °C at 20 °C/min and were held for 5 min at this temperature in order to remove any thermal history. Samples were then rapidly quenched to 135 °C at 80 °C/min and held isothermally for 100 min to record the time taken to reach the crystallization peak.

The crystalline structure of pure PP and the composites was studied by X-ray diffraction (XRD) analysis, which was carried out using the Rigaku SmartLab (Tokyo, Japan) with a CuKα radiation source. The X-ray was generated at 40 kV and 30 mA power, and XRD scans were recorded at 2θ from 5 to 90° (λ = 1.54 Å) at 3.04 deg/min. The percentage of β phase in the PP matrix was calculated using the $K_{\beta }$ equation:

(1) $$K_{\beta }=\frac{A_{\beta \left(300\right)}}{\sum A_{cryst}}$$

where $A_{cryst}$ is the area under each successive crystal peak, and $A_{\beta \left(300\right)}$ is the area under the peak of β(300).

The fiber length distribution in the composites was measured by pyrolyzing the PP matrix to extract fibers. Each specimen before the tensile test was wrapped in aluminum foil and then placed in a muffle furnace (Yamato, Tokyo, Japan, FP 410) at 500 °C for 30 min. The ash remaining on the aluminum foil was dispersed in pure water. The dispersion was dropped onto a Petri dish, covered with a cover glass, and photographed under an optical microscope. The lengths of at least 2700 fibers were measured.

The fracture surface of the specimen was observed using a scanning electron microscope (SEM). After the tensile test, specimens were coated with platinum (VACUUM DEVICE, MSP-1S, Ibaraki, Japan) and observed using an SEM (JEOL, Tokyo, Japan, JSM-6510LA) at 15 kV.

Crystallization near the fibers was observed using a polarizing optical microscope (POM, ECLIPSE L150, Nikon, Tokyo, Japan). Glass slides were placed on a hot stage set at 240 °C and warmed. The PP matrix incorporating GNPs and virgin fibers was sandwiched between the glass slide and cover glass, cooled to 130 °C, and held at 130 °C for 10 min.

## 3. Results and Discussion

### 3.1. Fiber Length Distribution

**Figure 1:**
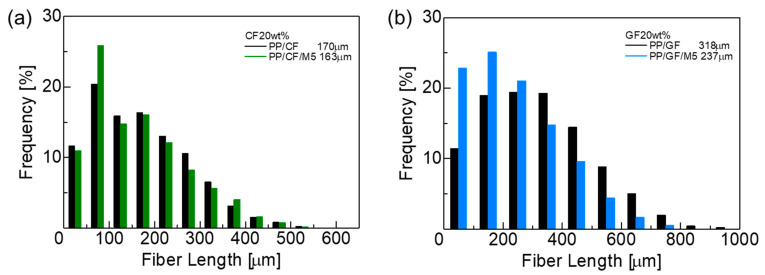
Fiber length distribution of (**a**) PP/CF and PP/CF/M5 and (**b**) PP/GF and PP/GF/M5 composites.

Fibers were extracted by pyrolyzing the matrix of the specimens before tensile testing, and the fiber length of each composite was measured using image analysis software. Figure 1 shows the fiber length distribution and average fiber length of the composites. As shown in Figure 1, the average fiber length was 170 µm for PP/CF and 318 µm for PP/GF. The average fiber length of both composites was shorter than the initial fiber length of 3 mm, suggesting breakage during the melt blending and injection molding processes. Considering the initial fiber length for both was 3 mm, CF tends to be shorter than GF, as CF is more brittle and fractures more easily than GF during processing [16]. The addition of GNPs decreased the average fiber length, decreasing it to 163 µm for PP/CF/M5 and 237 µm for PP/GF/M5. Adding GNPs seems to have increased the viscosity during melt blending, which may have caused the fibers to fracture more easily [28,34].

### 3.2. Mechanical Properties

**Figure 2:**
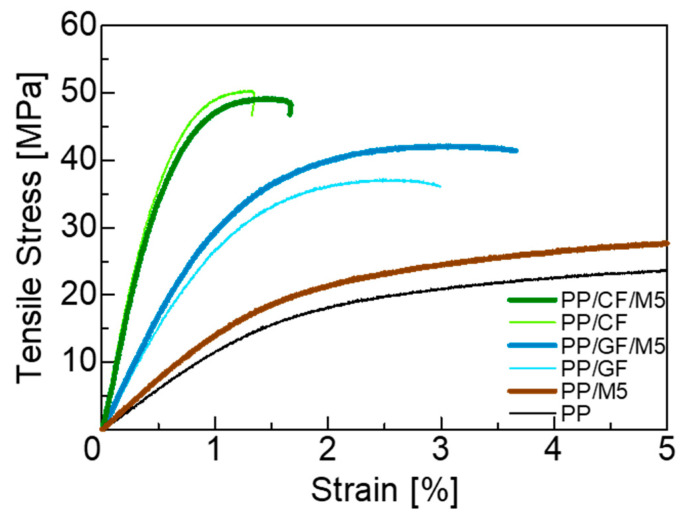
Tensile stress–strain curves of PP and PP composites.

The mechanical properties from the tensile test are presented in Figure 2 and Table 2. In general, incorporating fibers into polymers reduces fracture strain [16,43,44]. Composites reinforced by CF have a lower fracture strain than composites reinforced by GF. This is due to the difference in the modulus of elasticity of the fibers. Adding GNPs to PP/CF did not significantly change the mechanical properties, but adding GNPs to PP/GF increased the tensile strength by approximately 20% and the Young’s modulus by approximately 10%, despite the average fiber length being shorter compared to PP/GF. The modified rule of mixture can be used to calculate the elastic modulus of hybrid composites:

(2) $$E_{c}=\eta_{0}E_{f}V_{f}+E_{m}(1-V_{f})$$

where $E_{c}$ is the elastic modulus of the composite, $V_{f}$ is the volume fraction of filler, $E_{m}$ is the elastic modulus of the matrix, and $\eta_{0}$ is a correction factor. Using the PP/CF and PP/GF composites’ tensile test results, $\eta_{0}$ can be used to calculate the elastic modulus of the hybrid composites. The elastic modulus of the matrix in the hybrid composite $E_{m}$ uses the value of PP/M5. The theoretical elastic modulus of PP/CF/M5 and PP/GF/M5 can be calculated to be 8.83 GPa and 3.50 GPa, respectively. The elastic modulus of the PP/GF/M5 composite is close to the theoretical value, but the theoretical elastic modulus of the PP/CF/M5 composite is higher than the experimental value, suggesting GNP aggregation. The addition of GNPs had no effect on improving the mechanical properties, presumably due to the negative effect on residual fiber length, and no effect on interfacial adhesion properties [45].

The tensile strength of SFRPs can be calculated by the modified Kelly–Tyson model [25]:

(3) $$\sigma_{c}=\eta_{\theta }\left(\sum_{L_{i}<L_{c}}\left[\frac{\tau L_{i}V_{i}}{D}\right]+\sum_{L_{j}>L_{c}}E_{f}\varepsilon_{c}V_{j}\left[1-\frac{E_{f}\varepsilon_{c}D}{4L_{j}\tau }\right]\right)+\sigma_{m}\left(1-V_{f}\right)$$

where $\sigma_{c}$ and $\sigma_{m}$ represent the tensile strength of the composite and matrix, respectively; $L_{c}$ is the critical fiber length of the SFRP, with the subscripts i and j representing the subcritical and supercritical fibers, respectively; $V_{i}$ and $V_{j}$ represent the fiber volume fraction of the subcritical and supercritical fibers, respectively; τ is the IFSS between the fiber and matrix; D is the fiber diameter; $E_{f}$ is the fiber longitudinal modulus; $\varepsilon_{c}$ is the composite fracture strain; and $\eta_{\theta }$ is the fiber orientation factor. Wongpajan et al. [46] and Thomason et al. [47] revealed that the orientation factor of GF was around 0.59 when the GF content was 20 wt%. We calculated the fiber orientation factor for CF and GF as 0.59.

**Figure 3:**
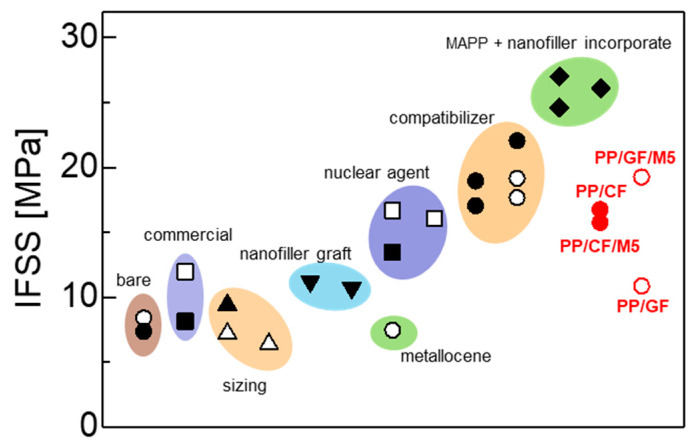
IFSS between CF and PP (full point) and between GF and PP (open point), as reported in the literature [17,28,36,48,49,50,51,52,53,54,55,56,57].

Here, we use Equation (3) and Table 3 to predict the IFSS. The predicted IFSS values for PP/CF and PP/CF/M5 were 16.7 MPa and 15.7 MPa, respectively. The IFSS between PP and CF here appears to be higher compared to values in the literature (8–9 MPa) [28,36,48,49,50], but the addition of GNPs did not change the IFSS very much. This result contradicts previous research that successfully incorporated nanofillers into PP to increase the IFSS between CF and the matrix [28], as well as previous research that incorporated GNPs to improve the mechanical properties of composites [34] (Figure 3). We assume that this is due to the difference in the carbon fibers used. In those studies, CFs with a rough fiber surface were used. The use of fibers with rough surfaces causes failure due to stress concentration in the grooves, but stress transfer may be improved in the presence of nanofillers. We consider that the use of CFs with smooth surfaces in this study limited the effect of nanofiller addition. A possible reason for the higher IFSS of PP/CF compared to other study results could be the ability of CFs to produce β phase crystalline structures (see Section 3.5). When β phase crystalline structures are produced at high densities at the interface, the IFSS increases [51]. On the other hand, the predicted IFSS values for PP/GF and PP/GF/M5 were 10.8 MPa and 19.2 MPa, respectively, and the addition of GNPs increased the IFSS by approximately 80%. The IFSS predicted for PP/GF is close to the literature value (τ=11.1 MPa) measured using the fragmentation test [17]. The IFSS (τ=19.2 MPa) predicted for PP/GF/M5 between GF and PP is equivalent to that when a compatibilizer such as MAPP is added to PP [52,53,54,55,56,57].

### 3.3. Fracture Surface

**Figure 4:**
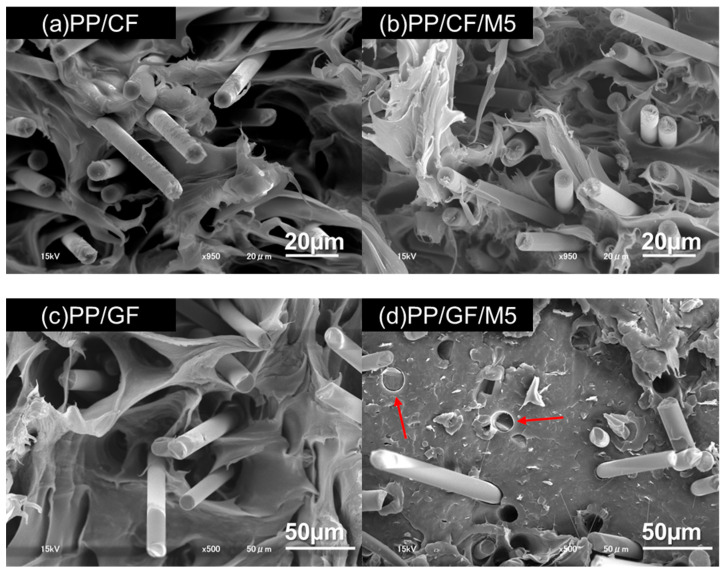
SEM images of fracture surfaces of (**a**) PP/CF, (**b**) PP/CF/M5, (**c**) PP/GF, and (**d**) PP/GF/M5 composites. Red arrows indicate fiber fracture at the fracture surface, which indicates strong interfacial bonding.

The failure mode of composites reinforced with fibers shorter than the critical fiber length is mainly due to fiber pull-out. The fracture mechanism of SFRPs during the tensile test was previously studied by Sato et al. [58]. They concluded that failure begins at the interface between the fiber end and the fiber side, leading to the propagation of interfacial cracks along the fiber side. Figure 4 shows the fracture surfaces of the tensile test specimens. The fracture surfaces of the PP/CF and PP/GF specimens showed ductile fracture with dimples [34]. This indicates that the reinforcing fibers in the composite were shorter than the critical fiber length. The amount of resin coverage on the fiber represents the interfacial interaction. Pulled CFs and GFs show smooth and clean surfaces, implying weak interfacial interaction. Furthermore, there is a gap between the fiber and the resin, which can be attributed to interfacial fracture. There was no significant difference in the appearance of the fracture surface between PP/CF and PP/CF/M5. However, the fracture surfaces of PP/GF and PP/GF/M5 were very different when comparing them. The PP/GF/M5 fracture surface has areas of fracture without dimples, and it can be observed that GNPs are dispersed in the matrix (Figure 4d). The SEM fracture surface image of PP/GF/M5 highlights where the fibers appear to have fractures (red arrows in Figure 4d), implying that the IFSS increased. However, there is still a gap between the GFs and the matrix, and the amount of resin coverage on the fibers is barely observed, indicating that there is still potential for the IFSS to increase. Sansone et al. [59] noted that GFs and GNPs bond chemically or electrostatically during melt blending or during the injection molding process, but there was no evidence of GNPs bonding to the GFs on this fracture surface.

### 3.4. Isothermal Crystallization

**Figure 5:**
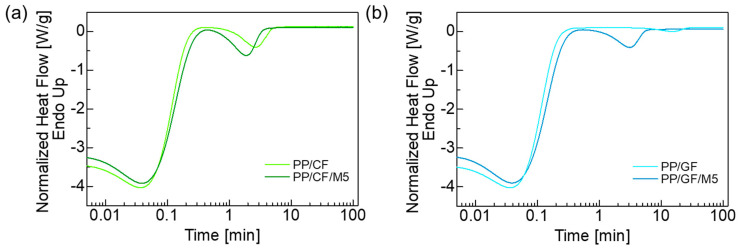
DSC isothermal crystallization curves of the (**a**) PP/CF and PP/CF/M5 and (**b**) PP/GF and PP/GF/M5 composites at 135 °C.

**Figure 6:**
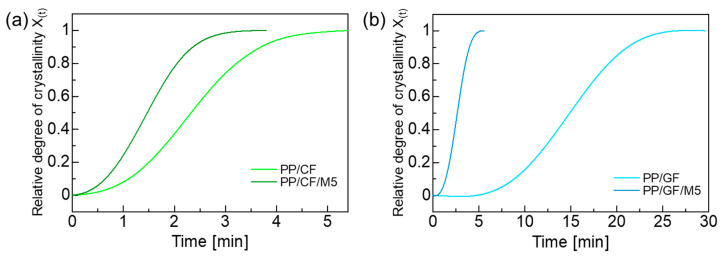
Relationship between relative degree of crystallinity *X*_(*t*)_ and isothermal crystallization time at 135 °C. (**a**) PP/CF and PP/CF/M5; (**b**) PP/GF and PP/GF/M5.

The results of the isothermal crystallization measurements at 135 °C using DSC are shown in Figure 5. According to the isothermal crystallization experiments, PP/CF crystallized faster than PP/GF, with a shorter time to peak crystallization. The peak crystallization time of the PP/CF/M5 and PP/GF/M5 composites containing GNPs decreased, indicating that the GNPs promote crystallization. From the results of the isothermal crystallization measurements, the relationship between relative crystallinity and crystallization time could be calculated using the following equation:

(4) $$X_{\left(t\right)}=\frac{\int_{0}^{t}\left(\frac{dH}{dT}\right)dT}{\int_{0}^{\infty }\left(\frac{dH}{dT}\right)dT}$$

where dH denotes the measured enthalpy of crystallization, and t and ∞ indicate the elapsed crystallization time and the end time of the crystallization phenomenon, respectively. Figure 6 shows the relative crystallinity of the composites during isothermal crystallization at 135 °C. The analysis of crystallization kinetics under isothermal conditions was performed using the Avrami equation:

(5) $$X_{\left(t\right)}=1-\exp\left[-{\left(Kt\right)}^{n}\right]$$

where $X_{\left(t\right)}$ is the relative crystallinity at time t, K is the crystallization rate constant, and n is the Avrami exponent. The values of K and n were determined by fitting the Avrami equation to the curve in Figure 6, and the results are listed in Table 4.

It was found that the crystallization rate constant *K* increased with the addition of GNPs. The Avrami exponent was about the same at about 2.4 for both PP/CF and PP/CF/M5, which means that CFs and GNPs can be heterogeneous nucleating agents [60,61]. On the other hand, *n* was larger than 3 for PP/GF, suggesting homogeneous nucleation from the PP matrix. This implies that GFs have no nucleation ability. The presence of GNPs in the PP/GF composite accelerated the crystallization rate, but crystal growth from GNPs was inhibited [38].

### 3.5. Crystalline Microstructure

**Figure 7:**
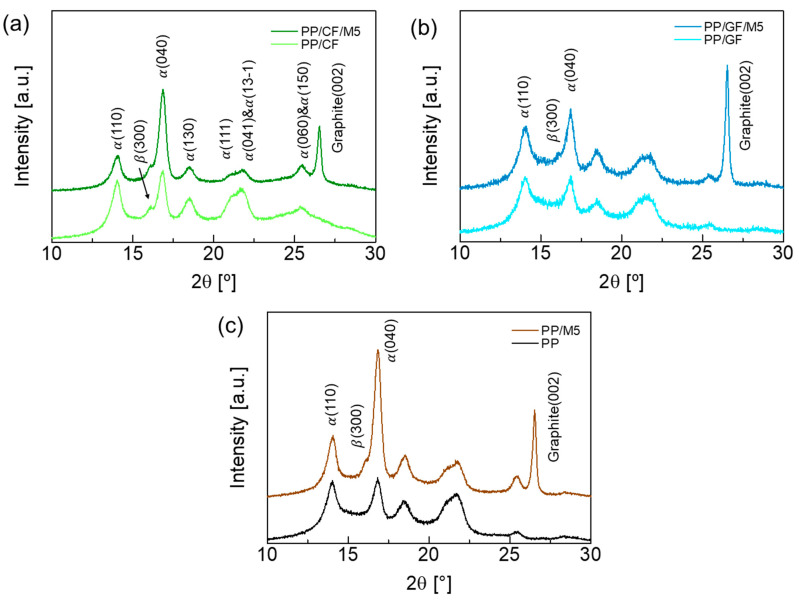
XRD spectra of refracted wavelengths at different 2θ angles: (**a**) PP/CF and PP/CF/M5, (**b**) PP/GF and PP/GF/M5, and (**c**) PP and PP/M5.

Crystal microstructure studies were conducted using XRD. Figure 7 shows the XRD patterns of PP and the PP composites. The nucleation mechanism of GNPs is believed to be epitaxial growth, which is caused by the alignment of the (002) plane of GNPs with the (004) plane of PP. Therefore, the nucleation efficiency is quantified by the peak intensity ratio of α(040) to α(110). The $I_{\alpha \left(040\right)}/I_{\alpha (110)}$ ratios of the composites are listed in Table 5. The $I_{\alpha \left(040\right)}/I_{\alpha (110)}$ ratio increased with the presence of CF and GNPs but not GF. CF and GNPs induced the β-form crystal and increased the $K_{\beta }$ value from 0.51 for PP to 1.66 and 4.64, respectively (Table 5) [41,62,63].

**Figure 8:**
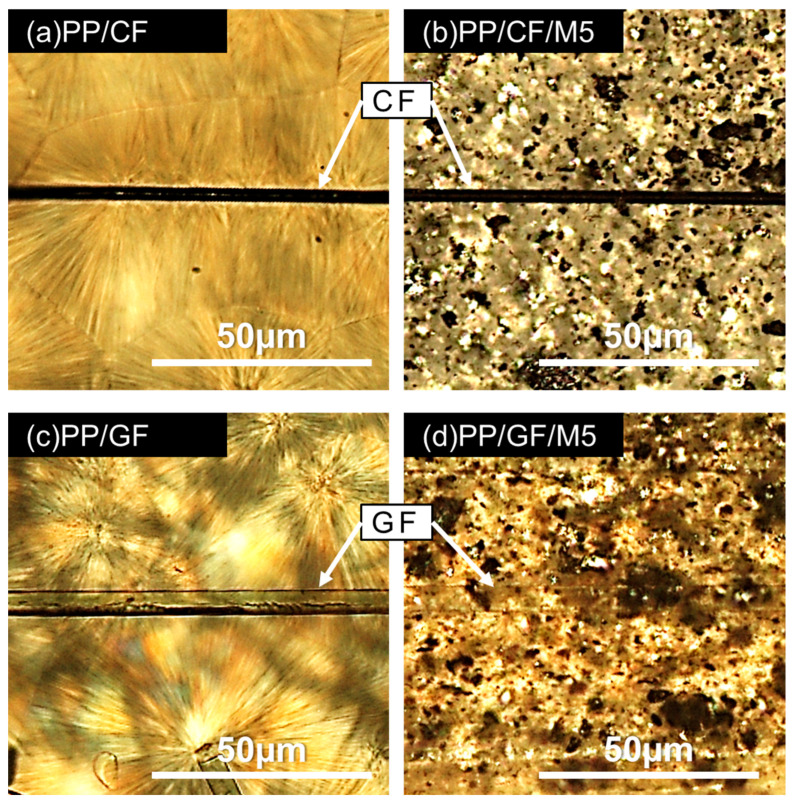
POM images of (**a**) PP/CF, (**b**) PP/CF/M5, (**c**) PP/GF, and (**d**) PP/GF/M5 crystallized after cooling from a molten state at 240 °C to 130 °C and holding isothermally at 130 °C for 10 min.

Figure 8 shows POM images of the samples crystallized after cooling from a molten state at 240 °C to 130 °C and holding isothermally at 130 °C for 10 min. The black or transparent area in the middle of the image is CF or GF. In Figure 8a, we can see crystals growing from the CF, but in Figure 8c, no crystals are growing from the GF. These results are consistent with the DSC and XRD results, indicating that CF is a nucleating agent, while GF is not. We can also see spherulites away from the CF and at PP/GF, with sizes as large as 50 µm in diameter. The addition of GNPs clearly reduced the crystal size, making it impossible to observe the crystal morphology in the vicinity of the fiber.

**Figure 9:**
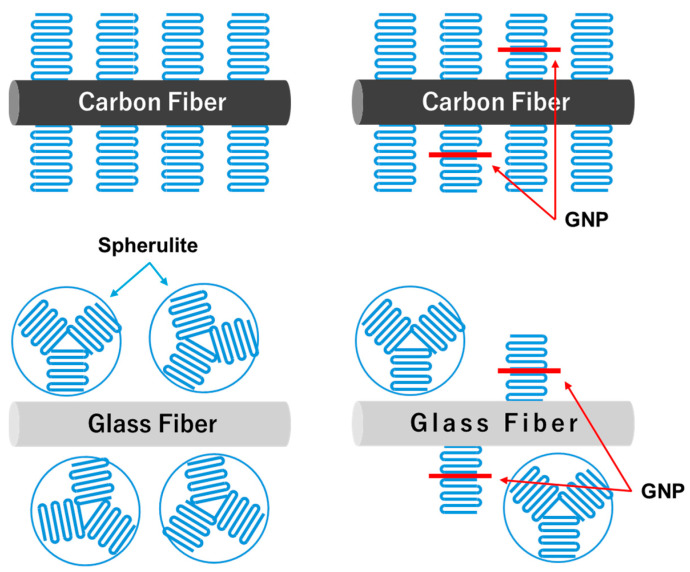
Schematic diagram of PP crystallization behavior of CF- and GF-reinforced PP composites with the addition of GNPs.

Based on the crystalline characterization, GNPs and CF can act as heterogeneous nuclear agents, but GF cannot. The presence of GNPs in PP/CF has little effect on crystal growth, but GNPs in PP/GF accelerate the crystallization rate, suggesting that the PP matrix behave spherulite growth in without GNPs, but when GNPs is present, it changes to epitaxial growth of PP crystals from GNPs. Figure 9 depicts the difference in crystal microstructure of the PP/CF or PP/GF specimens in the presence of GNPs.

## 4. Conclusions

In this study, we investigated the effect of adding a small amount (1 wt%) of GNPs to fiber-reinforced polypropylene composites on their mechanical properties and crystallinity microstructure. The mechanical properties characterization results showed no significant effect when GNPs were added to PP/CF, but when added to PP/GF, they increased the tensile strength and Young’s modulus by approximately 20% and 10%, respectively. We predicted the IFSS using the modified Kelly–Tyson model, a well-known model for predicting the strength of SFRPs. The addition of GNPs had little effect on the IFSS of PP/CF but increased the IFSS of PP/GF by approximately 80%. The tensile strength of the hybrid composite with GNPs added to PP/GF increased due to the increase in IFSS, even though the fiber length in the composite was decreased compared to PP/GF. The DSC results indicated that the GNPs accelerated the crystallization rate, and the XRD results confirmed that the GNPs acted as a crystal nucleating agent. CF is also a nucleating agent, but GF is not. The results of observations by POM show that the addition of GNPs reduced the crystal size of the PP matrix, suggesting that the crystalline morphology around the fiber would change. We concluded that different reinforcing fibers lead to different changes in mechanical properties when GNPs are added to composites. This method does not involve a complicated process and has potential for safe use, such as reducing the use of organic solvents, since GNPs are simply added together during melt blending.

## Figures and Tables

**Table 1:** Composition of polypropylene composites.

	PP [wt%]	CF [wt%]	GF [wt%]	GNP [wt%]
PP	100	-	-	-
PP/M5	99	-	-	1
PP/CF	80	20	-	-
PP/CF/M5	79	20	-	1
PP/GF	80	-	20	-
PP/GF/M5	79	-	20	1

**Table 2:** Tensile strength and Young’s modulus of each specimen.

	Tensile Strength [MPa]	Young’s Modulus [GPa]
PP	25.9 ± 0.15	1.24 ± 0.03
PP/M5	29.6 ± 0.21	1.54 ± 0.04
PP/CF	49.3 ± 1.93	8.57 ± 0.18
PP/CF/M5	50.1 ± 2.67	7.90 ± 0.16
PP/GF	36.4 ± 1.33	3.23 ± 0.11
PP/GF/M5	44.1 ± 1.17	3.54 ± 0.06

**Table 3:** Material parameters used for the calculation.

		PP/CF	PP/CF/M5	PP/GF	PP/GF/M5
Composite strength	$\sigma_{c}$	49.3 MPa	50.1 MPa	36.4 MPa	44.1 MPa
Fiber orientation factor	$\eta_{\theta }$	0.59			
Fiber strength	$\sigma_{f}$	4900 MPa		3200 MPa	
Average fiber length	L	170 µm	163 µm	318 µm	237 µm
Fiber volume fraction	$V_{f}$	0.11		0.08	
Fiber diameter	D	7 µm		13 µm	
Composite fracture strain	$\varepsilon_{c}$	0.015		0.03	
Elastic modulus of fiber	$E_{f}$	230 GPa		75 GPa	

**Table 4:** Isothermal crystallization parameters of PP composites.

	*K* [min^−n^]	*n*
PP/CF	0.0916	2.45
PP/CF/M5	0.275	2.47
PP/GF	0.0000631	3.44
PP/GF/M5	0.0375	3.00

**Table 5:** *I_α_*_(040)_/*I_α_*_(110)_ ratios and *K_β_* values of composites from XRD patterns.

	*I_α_*_(040)_/*I_α_*_(110)_	*K_β_* [%]
PP	0.898	0.51
PP/M5	3.064	4.64
PP/CF	0.981	1.66
PP/CF/M5	3.061	6.25
PP/GF	0.835	0.77
PP/GF/M5	1.186	4.14

## Data Availability

The original contributions presented in this study are included in the article. Further inquiries can be directed to the corresponding author.
